# Uveal myxoid leiomyosarcoma in a horse

**DOI:** 10.1002/ccr3.1190

**Published:** 2017-09-25

**Authors:** Siv Grosås, Liv Østevik, Tobias Revold, Nina Ottesen, Ernst‐Otto Ropstad

**Affiliations:** ^1^ Department of Companion Animal Clinical Sciences Faculty of Veterinary Medicine and Biosciences Norwegian University of Life Sciences Oslo Norway; ^2^ Department of Basic Sciences & Aquatic Medicine Faculty of Veterinary Medicine and Biosciences Norwegian University of Life Sciences Oslo Norway

**Keywords:** Equid, oncology, ophthalmology, veterinary

## Abstract

A uveal leiomyosarcoma of a horse is reported. There are few published reports of intraocular tumors in horses. Intraocular tumors challenge animal welfare by causing uveitis, glaucoma, and loss of vision. Knowledge regarding treatment of intraocular tumors with globe preservation is sparse, and further investigations on this topic are required.

## Introduction

Intraocular tumors are rare in the horse and can be primary or secondary. Primary intraocular tumors reported in the horse include melanoma, medulloepithelioma, and retinoblastoma, with melanomas and medulloepitheliomas being the most prevalent 1, 2, 3, 4, 5. Intraocular lymphomas are most frequently reported in association with multicentric forms of the disease, but a solitary primary ocular lymphoma has been described in a horse 6. All types of tumors can theoretically metastasize to the eye, with malignant lymphoma being the most common metastatic intraocular tumor across all species 5. The intraocular tumors’ space‐occupying nature in the globe might cause uveitis or blindness with limited treatment options for preserving vision. There are presently few published reports of intraocular tumors in horses. However, more cases are likely to be reported in the future as veterinary medicine develops and attention to veterinary ophthalmology increases.

## Case History/Examination

A 19‐year‐old warmblood horse was referred to the Norwegian University of Life Sciences for examination of the left eye. The referring veterinarian had observed a mass in the anterior chamber and described that the horse had shown signs of ocular discomfort with blepharospasm and excessive lacrimation from the left eye 4 days prior to referral. The duration of symptoms was estimated by the owner to be 14 days. There was no known history of trauma or other disease, and the horse's general health condition was unremarkable. There were no abnormal findings at clinical examination of the horse apart from the left eye.

**Figure 1:**
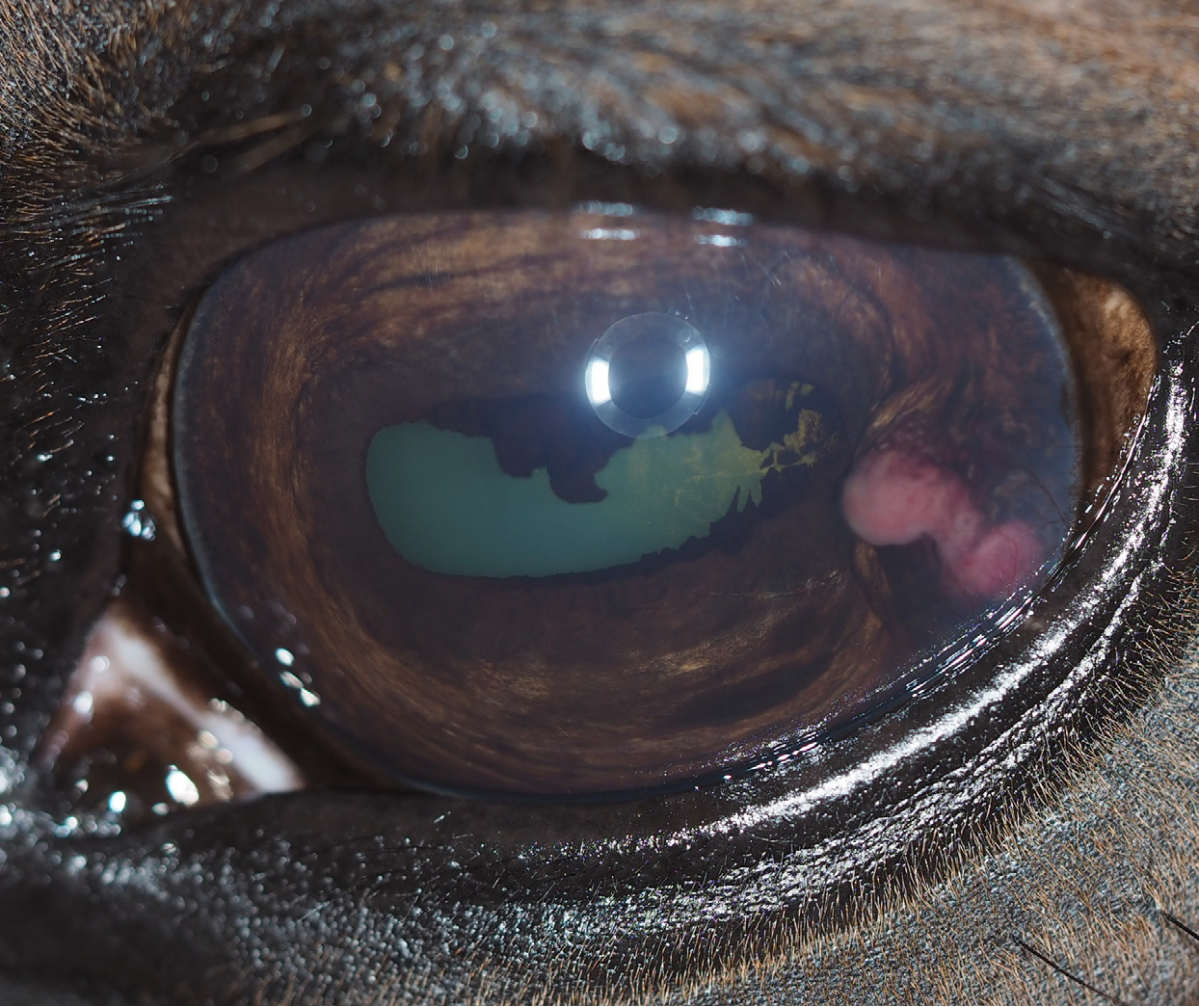
The clinical appearance of the left eye at presentation. A pink, nonpigmented, rounded, and partially lobulated mass is seen at the temporal and anterior surface of the iris. The mass is extending into the anterior chamber partially located against the corneal endothelium in the temporal and ventral corneal quadrant, where corneal neovascularization is present. Posterior synechiae and cortical equatorial cataract are visible at the temporal margin of the iris and lens.

A complete ophthalmic examination with a slit‐lamp biomicroscope and an indirect ophthalmoscope with a 20 D lens was performed. Palpebral reflexes and menace response were normal in both eyes. The pupil of the left eye was mildly miotic, while the pupillary reflexes were otherwise normal. No prominent blepharospasm or excessive lacrimation was present. Examination of the left eye revealed a pink, nonpigmented, rounded, and partly lobulated mass arising from the ventrolateral anterior surface of the iris, extending into the anterior chamber, partially touching the corneal endothelium in the ventral and lateral corneal quadrant (Fig. 1). The corneal surface was smooth and the corneal clarity was considered normal, except from slight corneal edema and neovascularization corresponding to the mass (in the ventrolateral quadrant of the cornea). Conjunctiva and episclera displayed a mild vascular congestion. The iris was slightly darker pigmented than iris of the right eye, and posterior synechiae were present in the area of the mass. Mild aqueous opacification/flare in the anterior chamber was present, and a cortical equatorial cataract corresponding to the posterior synechiae was noted. The lens was otherwise mildly sclerotic and situated in its normal position. Fundic examination was unremarkable. The right eye and adnexa were considered normal. Schirmer tear test was 22 and 25 mm/min in the right and left eye, respectively. Intraocular pressure was measured to be 21 mmHg in the right eye and 12 mmHg in the left eye using a rebound tonometer (Icare^®^ TONOVET, Icare Finland Oy, Vantaa, Finland). The recordings were consistent with a normal pressure in the right eye and a decreased pressure in the left eye, consistent with uveitis. Fluorescein‐Na staining was negative in both eyes.

The ocular ultrasonographic examination was conducted using GE Healthcare Logiq E9 ultrasound machine. The eye was examined using a trancorneal approach making use of linear array multifrequency probes (9L‐D and ML6‐15‐D) and sterile acoustic coupling gel.

**Figure 2:**
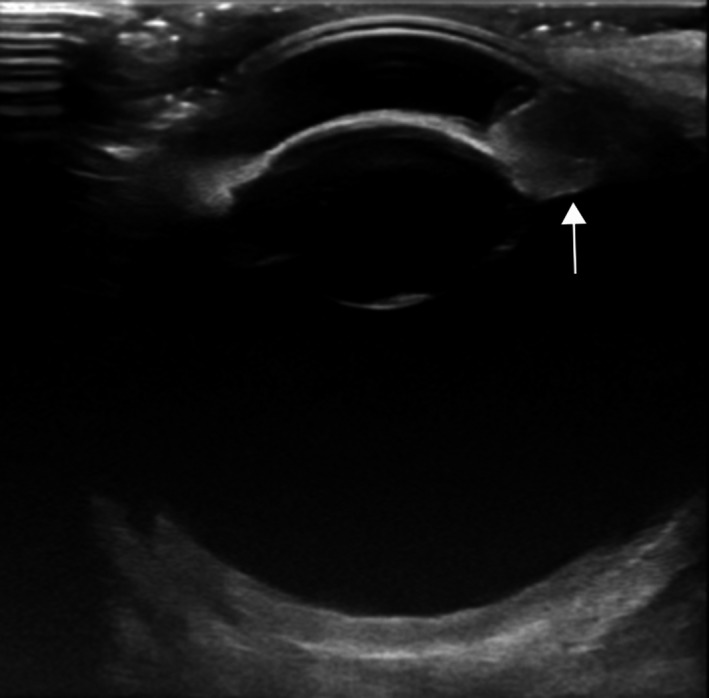
Transversal transcorneal ultrasonography of left eye shows an uveal echogenic mass extending into anterior chamber (arrow).

The examination was performed after sedation of the horse and application of a topical anesthetic, oxybuprocaine hydrochloride 0, 4% (Oxibuprokain Minims, Bausch & Lomb, London, Great Britain UK). The bulbar structures and cornea had a normal ultrasonographic appearance. A rounded, smooth, well defined and homogenous echoic mass originating from the ventrolateral aspect of iris extending into the anterior chamber was identified (Fig. 2). The mass measured 8 mm in diameter and showed moderate vascularization performing power Doppler imaging. The lens contained a thin echoic line compatible with early cataract identified at the slit‐lamp examination. The retina and vitreous were considered to be within normal limits.

## Differential Diagnosis, Investigations, and Treatment

In summary, clinical findings were consistent with an iridial mass of unknown malignancy, with associated mild uveitis. A medulloepithelioma, an amelanotic melanoma, or a lymphoma were considered the most likely differentials based on earlier reports. However, other mesenchymal or epithelial tumors could not be excluded as differentials, and enucleation with histopathological examination to achieve an exact diagnosis and prevent further extension of the tumor into extraocular tissues was advised.

Enucleation was recommended for diagnostic and therapeutic purposes. Palliative treatment of the uveitis with topical cyclopentolate hydrochloride 1% (Cyclopentolat^®^, Bausch & Lomb, London, Great Britain UK) BID, diclofenac 0, 1% (Voltaren Ophtha^®^, Thea Laboratoires, Clermont‐Ferrand, France) TID a neomycin/polymyxin B sulfate/dexamethasone ophthalmic solution (Maxitrol^®^, Alcon Laboratories Inc, Freiburg, Germany) QID, and meloxicam suspension (Metacam vet^®^, Boehringer Ingelheim Vetmedica GmbH, Ingelheim am Rhein, Germany) per os SID was administered until scheduled enucleation 6 days later. A transconjunctival enucleation was performed, and the eye was fixed in 4% unbuffered formalin.

**Figure 3:**
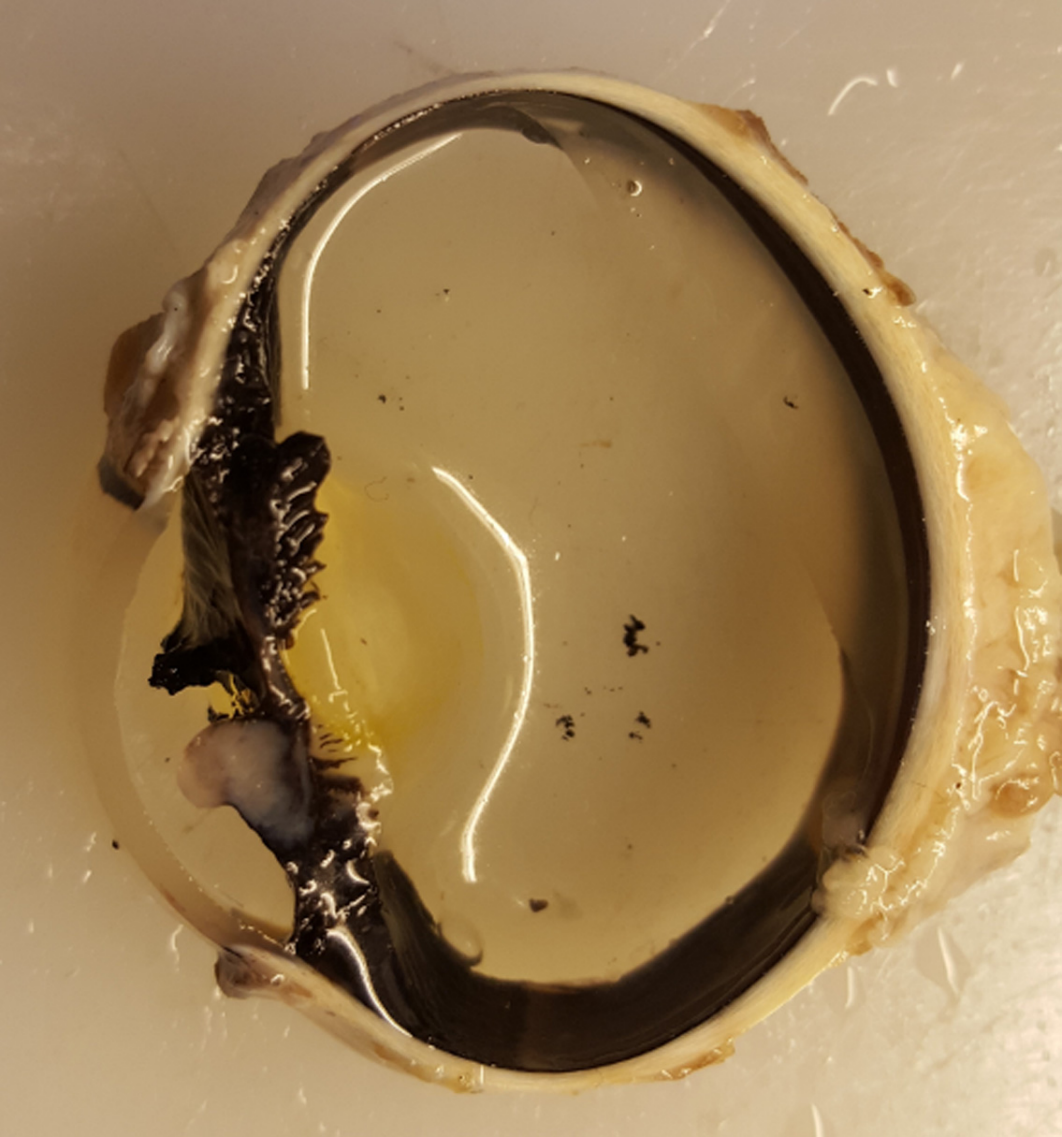
Parasagittal section of the eye. A white, approximately 5 × 8 mm, nonencapsulated, and infiltrative tumor expands the iris.

The eye was transected in the sagittal plane just medial to the optic nerve, and a second lateral parasagittal cut was made transecting the mass expanding the iris. The mass was white, approximately 5 × 8 mm in diameter, nonencapsulated, and infiltrative and had a solid, homogenous cut surface (Fig. 3). Axially, the mass was adherent to the lens. Tissue was routinely processed, embedded in paraffin, sectioned at 1–2 microns, and stained with hematoxylin and eosin for histological examination. Additionally, staining with Congo red and Alcian blue was performed on selected tissue blocks.

**Figure 4:**
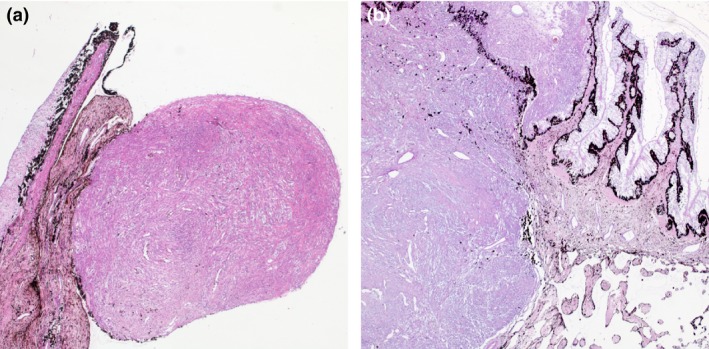
Histology, low magnification (A and B). (A) An apparently well‐demarcated, cell‐rich, mesenchymal tumor is protruding into the anterior chamber. (B) However, section through another part of the tumor reveals infiltrative growth and tumor tissue replacing and expanding the ciliary body and ciliary processes. Hematoxylin and eosin stain, 2.5x objective.

**Figure 5:**
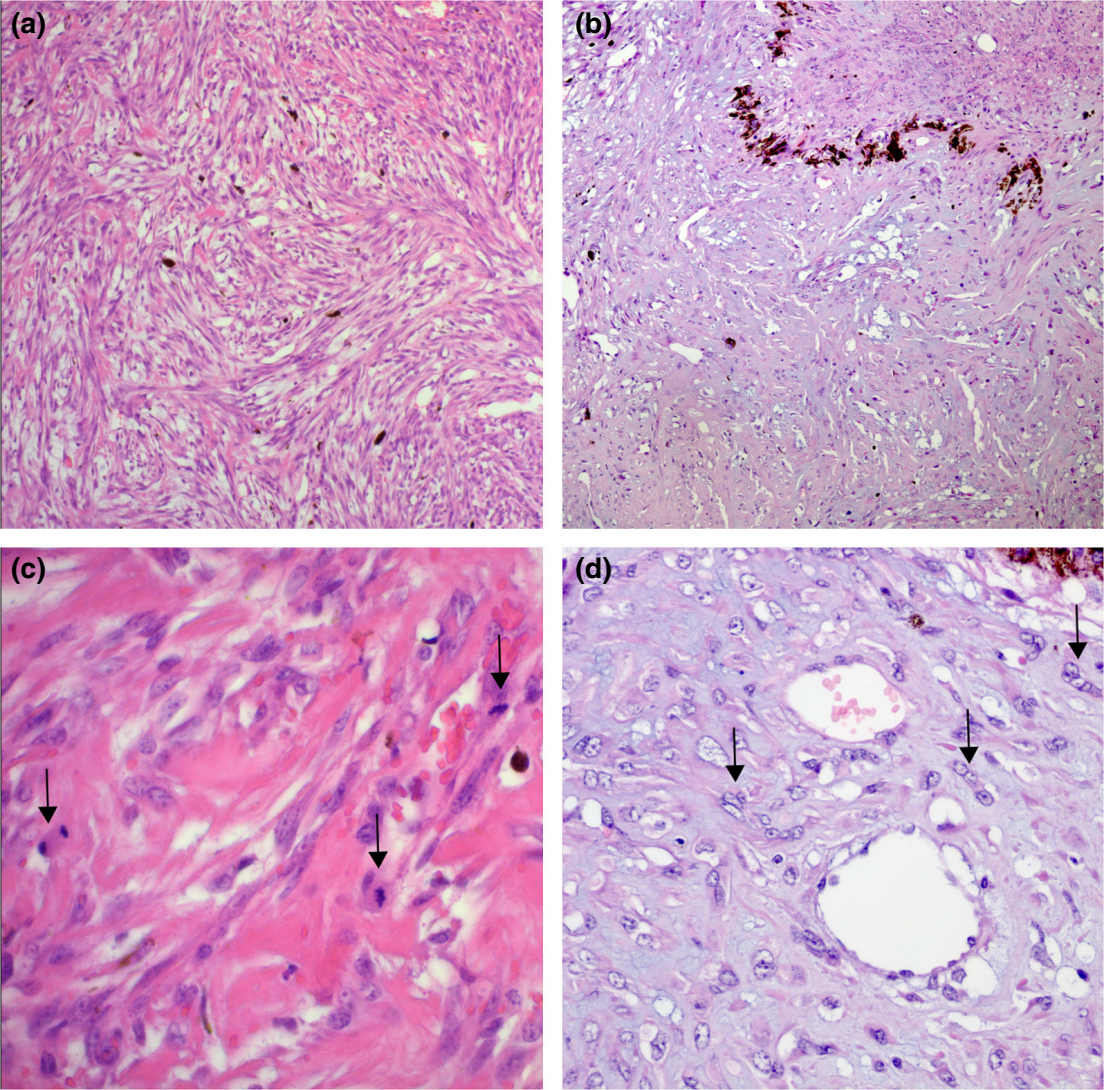
Histology, medium (A and B), and high magnification (C and D). (A) Slender neoplastic spindle cells form streams and bundles. (B) Small to marked amounts of basophilic mucinous material separate individual or bundles of tumor cells. Hematoxylin and eosin stain, 10x objective. (C–D) Tumor cells have centrally located, oval, basophilic nuclei with vesicular chromatin and 1–3 nucleoli, moderate amounts of eosinophilic cytoplasm, and indistinct borders. (C) Mitotic figures were relatively frequent (arrows), and (D) multinucleated cells were present (arrows). Hematoxylin and eosin stain, 40x objective.

**Figure 6:**
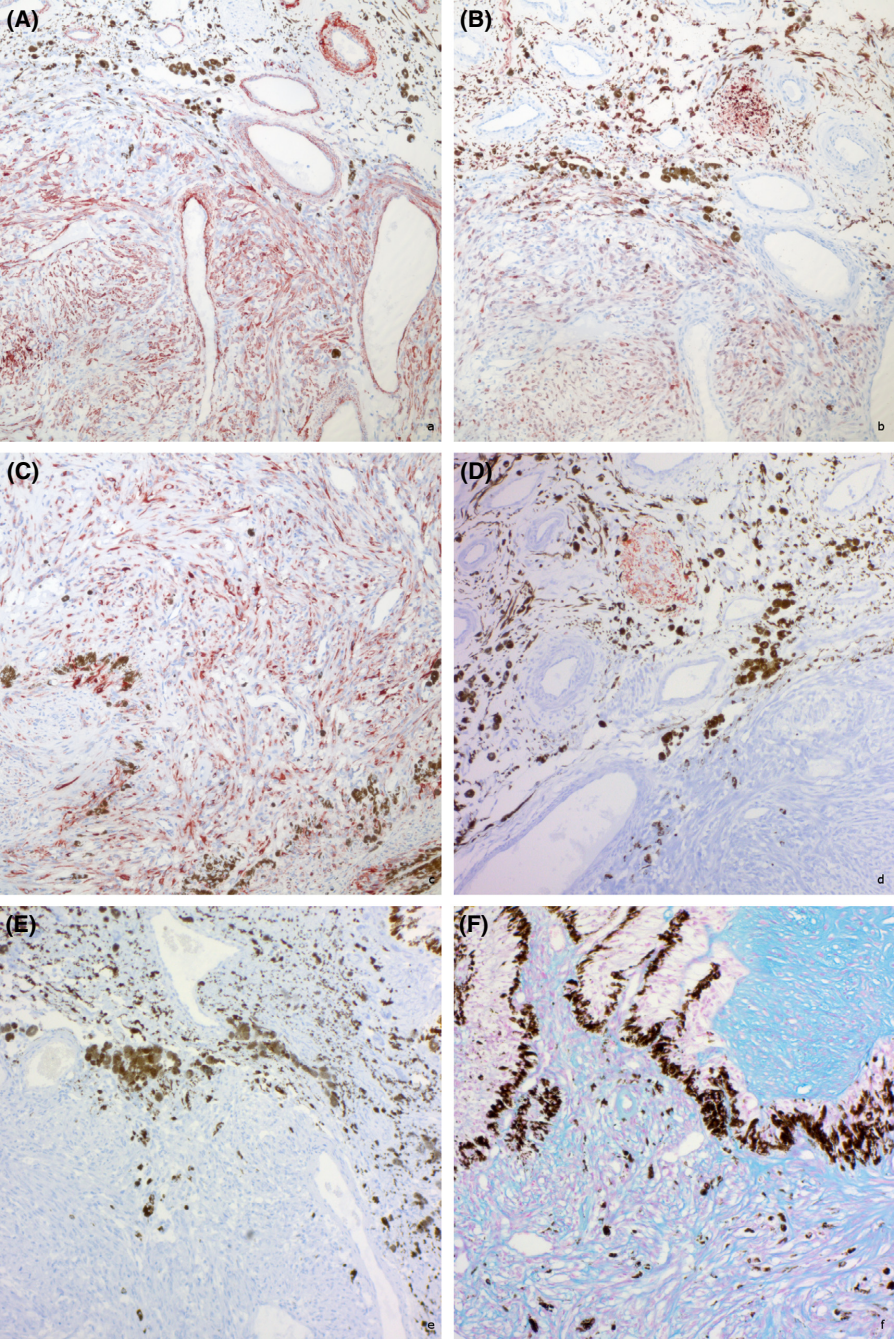
Immunohistochemistry (A–E) and alcian blue stain (F). (A) The majority (>60%) of tumor cells show strong cytoplasmic signal for SMA. Note internal positive control in vascular smooth muscle. (B) Around 50–60% display moderate cytoplasmic immunopositive staining for S100. Note strong immunostaining of nerve tissue in the iris. (C) Tumor cell cytoplasm stain strongly with desmin, but staining varies from 30% to 60% of cells. Notably, the nonpigmented ciliary epithelium also displays strong positive cytoplasmic desmin staining. (D) All neoplastic cells were negative for GFAP, while nerve tissue in the iris stain positive. (E) Neoplastic cells are consistently negative for PNL2; however, no staining for PNL2 was observed in any cell in the ciliary body or iris. Immunohistochemistry, 10x objective. (F) The matrix of the tumor stained positive with alcian blue consistent with presence of mucopolysaccharides. Alcian blue 10x objective.

Histological examination revealed the growth of a nonencapsulated, solid, infiltrative, cell‐rich mesenchymal tumor expanding and replacing the iris and ciliary processes (Fig. 4A and B). Slender spindle cells with moderate amounts of eosinophilic cytoplasm and indistinct cell borders grew in streams and bundles. Bundles and single cells were surrounded by mild‐to‐moderate amounts of fibrous stroma. In the majority of the tumor, small to marked amounts of basophilic mucinous material separated individual and bundles of tumor cells (Fig. 5A and B). This material stained blue with the alcian blue staining (Fig. 6). The cells had centrally located, oval, basophilic nuclei with vesicular chromatin and 1–3 nucleoli, and multifocal binucleate and multinucleated cells were observed. Mitotic index was 20 per 10 high power field and the cells displayed marked anisokaryosis and moderate anisocytosis (Fig. 5C and D). Few round or spindle‐shaped pigmented cells were found entrapped within the tumor tissue. Focally, the tumor tissue was confluent with and adherent to the anterior lens capsule and the endothelium lining Descemet's membrane. A diagnosis of a malignant mesenchymal tumor was made. Fibrosarcoma, leiomyosarcoma, peripheral nerve sheath tumor, or amelanotic melanoma were considered possible differential diagnoses, and immunohistochemistry was deemed necessary to make a conclusive diagnosis.

Immunohistochemistry (IHC) was performed using the Envision method with antibodies against smooth muscle actin (SMA), glial fibrillary acidic protein (GFAP), S100, desmin, and the melanoma antigen PNL‐2. Source, dilution, antigen retrieval method, and incubation time for each primary antibody are available in Table 1. Antigen retrieval was followed by incubation with primary antibodies, secondary antibodies, and finally application of 3‐amino‐9‐ethylcarbazole (AEC) chromogen (Dako). Slides were counterstained with hematoxylin, dehydrated, and mounted under polyvinyl alcohol mounting medium. Positive controls consisted of equine tissue collected from unrelated horses (Table 1). For negative controls, addition of the primary antibody was omitted.

**Table 1:** Primary antibodies, source, dilution, incubation time, temperature, and positive controls

Antigen	Source	Primary antibody	Catalogue number	Dilution	Pretreatment	Incubation time (min)	Positive control
GFAP	Dako	Polyclonal rabbit anti‐bovine GFAP	Z0334	1:500	None	60	Cerebrum
PNL2	Santa Cruz Biotechnology, Inc	Monoclonal mouse anti‐human synthetic melanoma	sc‐59306	1:150	Autoclave/citrate buffer	30	Cutaneous melanoma
S100	Dako	Polyclonal rabbit anti‐bovine S100	Z0311	1:500	Trypsin	60	Cerebrum
Desmin	Dako	Monoclonal mouse anti‐human desmin	M0760	1:500	Microwave/citrate buffer	30	Myocardium
SMA	Dako	Monoclonal mouse anti‐human actin 1A4	M0851	1:100	Trypsin	60	Small intestine

The tumor cells (>60%) were strongly positive SMA and moderately positive for S100 (50–60%), while all neoplastic cells were negative for GFAP and PNL‐2. Tumor cells stained strongly with desmin, but staining varied from approximately 30–60% of the cells (Fig. 6). Notably, the nonpigmented ciliary epithelium also displayed strong positive cytoplasmic desmin staining. Results of the immunohistochemistry combined with the growth pattern of the tumor were thus considered most consistent with a uveal leiomyosarcoma. Additional findings included neutrophilic keratitis and conjunctivitis with corneal edema and peripheral neovascularization, lymphohistiocytic uveitis, lenticular cataract, multifocal loss of retinal ganglion cells, and focal retinal detachment. Mild amounts of eosinophilic amorphous material were covering the nonpigmented epithelium of the ciliary body. This material was negative for Congo red, and no intracytoplasmic inclusions or infiltrates of lymphocytes were found in the nonpigmented ciliary epithelium, thus excluding a diagnosis of concomitant equine recurrent uveitis.

## Outcome and Follow‐up

Healing post enucleation was uneventful. The horse showed no signs of systemic disease or ocular disease in the remaining eye during the following 6 months.

## Discussion

A unilateral anterior uveal leiomyosarcoma, affecting the iris and the ciliary body of a horse, is reported. An iridal neoplasm was strongly suspected based on the ophthalmological examination; however, histopathology and immunohistochemistry were necessary to reach a final diagnosis. The diagnosis was made based on the neoplastic spindle‐shaped cells and the growth pattern combined with features of malignancy including infiltrative growth, relatively high mitotic index, and atypia. In addition, mucinous matrix separating the neoplastic cells was present in large proportions of the tumor. Strong cytoplasmic staining with SMA and desmin and no staining with GFAP confirms smooth muscle origin. Due to the extensive involvement of both iris and ciliary body, it was considered impossible to decide whether the tumor originated from the iridal or ciliary smooth muscle. The general health condition of the horse was unremarkable, and anamnestic or follow‐up information gave no indication of metastatic disease. A primary uveal myxoid leiomyosarcoma was thus considered the most likely diagnosis in this case.

Benign or malignant mesenchymal or epithelial tumors were considered possible differentials after clinical examination of the horse. As melanomas are among the most commonly reported intraocular tumors in horses 7, and as they most often develop from the iris or the ciliary body 1, 5, an amelanotic melanoma was considered one of the most likely diagnosis at the initial examination of the horse. A medulloepithelioma was also considered likely, due to several reports of horses with visual similarity to this case, presenting with a mass in the anterior chamber and accompanying signs of uveitis or glaucoma 3, 4. Iridociliary epithelial tumors have to the authors’ knowledge not been reported in the horse and were considered less likely. A granulomatous growth in response to a chronic inflammation with or without the presence of a foreign body was discussed initially, but considered less likely due to the absence of a puncture wound or other signs of trauma. The possibility of a cystic lesion was considered, but was excluded due to a solid appearance of the mass. Fibrosarcoma, leiomyosarcoma, peripheral nerve sheath tumor, and amelanotic melanoma were considered possible differential diagnoses after histological examination, and immunohistochemistry was necessary to reach a conclusive diagnosis.

Immunopositive staining with SMA and desmin, combined with lack of staining with GFAP, was consistent with a tumor of smooth muscle origin. Peripheral nerve sheath tumors in dogs are reported to be inconsistently positive for GFAP and S100, but are consistently negative for *α*‐SMA 8. In a study of schwannomas in 22 horses, all tumors stained positive for S100, while six of six tumors stained with GFAP were positive. No staining for SMA was performed 9. Equine melanomas were recently shown to stain consistently positive for PNL2, and this marker was also highly specific for equine melanocytic neoplasms 10. However, in the current case, no PNL2 expression was observed in any cells in the ciliary body or iris. To our knowledge, no reports regarding PNL2 expression of the normal equine ocular tissues or equine ocular melanomas exist, so whether lack of staining is related to factors specific to this case (e.g., fixation) or is equine uveal melanocytes really are PNL2 negative remains uncertain. In addition to leiomyomas and leiomyosarcomas; perivascular wall tumors, including hemangiopericytomas; are immunopositive for SMA. However, these tumors usually arise from the skin and subcutis and display characteristic growth patterns not observed in this case 11, 12. The positive staining with S100 was unexpected, as leiomyomas and leiomyosarcomas are reported to be negative for this marker. However, smooth muscle cells of the iris are derived from neural ectoderm, while the smooth muscle of the ciliary body is of neuronal crest origin, that is mesectoderm, and this may explain the S100 positive staining in this case 13. Supporting the notion of neural crest origin of ocular tissue, a substantial proportion of feline restrictive orbital myofibrosarcomas are also S100 positive 14.

Leiomyosarcomas are common primary tumors of the urogenital tract and the gastrointestinal tract in dogs and cats and are reported occasionally at these locations in several species including horses and cattle 15, 16, 17, 18, 19. Leiomyomas and leiomyosarcomas may develop in any organ containing smooth muscle cells including the smooth muscle cells of the iris and ciliary body. The smooth muscle cells of the iris and ciliary body are different in origin; the smooth muscle cells of the iris being embryologically derived from the neuroectoderm, while the smooth muscle cells of the ciliary body derive from the neural crest 20. Overall, there are few descriptions of intraocular tumors of smooth muscle origin in the veterinary literature. Intraocular leiomyoma originating from the iris has been reported as a rare primary intraocular tumor in dogs, and an intraocular leiomyosarcoma considered to be of iris dilator muscle origin was reported in a cat 21, 22. To the author's knowledge, there are no previous reports of intraocular leiomyosarcomas in the horse.

Soft tissue sarcomas are an important and frequently reported group of tumors in dogs. The malignant potential of soft tissue sarcomas varies to a great extent depending on the type of sarcoma and tissue location. Although distant metastases may occur, soft tissue sarcomas are in many cases stated to have a biological behavior characterized by local invasiveness rather than hematogenous metastasis 23. Regarding metastatic tumors reported in the globe and orbit across species, carcinomas predominate. This fact is suggested to be a consequence of carcinomas being more likely to undergo hematogenous dissemination than sarcomas 5.

Leiomyosarcomas of the urogenital‐ and the gastrointestinal tract, with or without local invasiveness, and multicentric presentation have been reported in horses 15, 24, 25, 26.

As there are no previous reports of intraocular leiomyosarcomas in the horse and limited literature on ocular tumors in general, the biological behavior of intraocular leiomyosarcomas in equids, the risk of extraocular invasiveness, and distant metastasis remains unclear. There were no signs of local invasiveness in the surrounding extraocular tissue or any suspicion of metastatic disease in the current case.

Primary intraocular tumors are often followed by no or only mild clinical signs of ocular disease early in the development of the disease. Thus, the diagnosis of an ocular tumor is often not made until a mass is macroscopically visible in the anterior segment of the eye, or before space‐occupying effects of the tumor cause symptoms of secondary ocular disease, such as uveitis, glaucoma, or blindness. Lack of intraocular inflammation upon clinical examination is suggested to be a characteristic presentation of primary intraocular tumors 2. However, the mild uveitis in this case was considered to be a result of the tumor's space‐occupying effects in the anterior chamber and compression against the cornea, leading to keratitis, as a plausible cause of a reflex uveitis. Furthermore, inflammation is always present to some extent in association with neoplastic growth. The degree of inflammation varies, but from a pathological point of view, virtually, all primary as well as secondary tumors are inflamed, with leukocyte infiltration being a hallmark neoplastic growth 27.

Primitive neuroectodermal tumors in horses originating from the iris and ciliary body have been suggested to have a more benign nature and better prognosis than those arising from the posterior uvea and the optic nerve 2, 28. Despite this, extraocular extension and metastatic spread have been reported from a medulloepithelioma of the ciliary body, suggestive of an unpredictable biological behavior of these tumors 4. In general, early enucleation is associated with a good prognosis for solitary iridal tumors, while involvement of the posterior uvea or optic nerve gives a guarded prognosis, and a more radical therapeutical approach including exenteration is often advised 2.

Fine‐needle biopsy is a possible diagnostic approach to differentiate between different types of intraocular tumors 29. However, this procedure is associated with a significant risk of intraocular hemorrhage and exacerbation of intraocular inflammation. Surgical removal of intraocular tumors by iridectomy or other techniques is also associated with a significant risk of complications. As ultrasound showed no involvement of the posterior segment of the eye, in this case, a standard transconjunctival enucleation was performed. The malignant potential and biological behavior of the tumor were unknown at the time of enucleation. Therefore, a guarded prognosis regarding recovery and function of the horse was given.

Enucleation or exenteration is, like in this case, often the treatment of choice when intraocular tumors are identified and the intended use and temper of the horse allows it. Most horses cope very well with one eye, but in some cases, enucleation is not advisable due to pronounced adrenergic or shy behavior. In this case, the horses temper or function was not a concern, as it was a calm horse used for pleasure purposes.

In general, intraocular tumors challenge animal welfare across species by causing uveitis and glaucoma with associated pain and ultimately loss of vision. Knowledge regarding response to more advanced treatment of intraocular tumors with globe preservation is sparse, and further investigations on this topic are required as life expectancy in horses as other companion animals are increasing.

## Authorship

SG, TR, EOR: designed the concept of the clinical case report and performed the clinical examination, treatment, and follow‐up of the patient, while LØ performed the pathological procedures. NO performed the ultrasonographic examination. All authors contributed with the writing and approval of the final manuscript.

## Conflict of Interest

The authors declare that they have no competing interests.
